# METTL3-dependent N6-methyladenosine modification is involved in berberine-mediated neuroprotection in ischemic stroke by enhancing the stability of NEAT1 in astrocytes

**DOI:** 10.18632/aging.205369

**Published:** 2024-01-04

**Authors:** Junya Hu, Huijie Duan, Junqing Zou, Wangli Ding, Ziqiao Wei, Qiang Peng, Zhongyuan Li, Rui Duan, Jianguo Sun, Junrong Zhu

**Affiliations:** 1 Department of Pharmacy, Nanjing First Hospital, China Pharmaceutical University, Nanjing 210006, China; 2 Department of Pharmacy, Nanjing First Hospital, Nanjing Medical University, Nanjing 210006, China; 3 Department of Neurology, Nanjing First Hospital, Nanjing 210006, China; 4 Key Lab of Drug Metabolism and Pharmacokinetics, State Key Laboratory of Natural Medicines, China Pharmaceutical University, Nanjing 210009, China; 5 Department of Second Clinical Medical School, Nanjing Medical University, Nanjing 210000, China

**Keywords:** ischemic stroke, m6A, berberine, Nampt, astrocytes

## Abstract

Ischemic stroke (IS) is one of the principal causes of disability and death worldwide. Berberine (BBR), derived from the traditional Chinese herbal medicine Huang Lian, has been reported to inhibit the progression of stroke, but the specific mechanism whereby BBR modulates the progression of ischemic stroke remains unclear. N6-methyladenosine (m6A) modification is the most typical epigenetic modification of mRNA post-transcriptional modifications, among which METTL3 is the most common methylation transferase. During the study, the middle cerebral artery occlusion/reperfusion (MCAO/R) was established in mice, and the mice primary astrocytes and neurons induced by oxygen-glucose deprivation/reoxygenation (OGD/R) was simulated *in vitro*. Level of LncNEAT1, miR-377-3p was detected via RT-qPCR. The levels of Nampt and METTL3 were measured by Western blot. CCK8 and LDH assay was performed to detect cell viability. Here, we found that berberine alleviates MCAO/R-induced ischemic injury and up-regulates the expression of Nampt in astrocytes, miR-377-3p inhibits the expression of Nampt in astrocytes after OGD/R, thus promoting neuronal injury. NEAT1 binds to miR-377-3p in OGD/R astrocytes and plays a neuronal protective role as a ceRNA. METTL3 can enhance NEAT1 stability in OGD/R astrocytes by modulating m6A modification of NEAT1. Taken together, our results demonstrate that berberine exerts neuroprotective effects via the m6A methyltransferase METTL3, which regulates the NEAT1/miR-377-3p/Nampt axis in mouse astrocytes to ameliorate cerebral ischemia/reperfusion (I/R) injury.

## INTRODUCTION

Ischemic stroke (IS) is an emergency cerebrovascular disease that accounts for 80% of all stroke and has the high morbidity, disability and mortality rate [1]. Thrombolytic therapy is currently perceived as the most potent treatment for stroke, and intravenous tissue plasminogen activator (tPA) is the uniquely validated cure for IS. However, tPA has a narrow therapeutic window and safety issues such as neurotoxicity and cerebral hemorrhage [2], thus few patients benefit. Accordingly, there is an urgent necessity to explore new therapeutic strategies, especially therapeutic agents linked to neuroprotection.

Nicotinamide phosphoribosyl transferase (Nampt) is the velocity-limiting enzyme of the mammalian NAD (nicotinamide adenine dinucleotide) biosynthetic compensation pathway [3]. It has been found that Nampt reduces the infiltration of neutrophils into the peri-ischemic area of the brain [4], and Zhao et al. found that the neuroprotective effect of extracellular visfatin against ischemic stroke was associated with its Nampt-related enzymatic activity [5]. Taken together, these point that Nampt is a target for the prevention and treatment of ischemic stroke. Therefore, the purpose of this study was to clarity natural active substances related to the Nampt target for the prevention and treatment of ischemic stroke and to investigate the mechanisms involved.

MicroRNAs are endogenous non-coding single-stranded RNAs of about 20 nt that adversely adjust the expression of target genes after transcription by pairing with complementary sequences in the 3’-UTR of the target gene. Recently, diverse miRNAs have been described to be present in neurological disease progression, including stroke. For example, inhibition of miR-182 protects against experimental stroke and attenuates astrocyte injury [6]. miR-98 reduces endothelial dysfunction and ameliorates ischemic /reperfusion injury in mice by protecting the blood-brain barrier [7]. MiR-34a-mediated SIRT1/mTOR signaling pathway attenuated d-galactose-induced brain senescence in mice [8]. It has been reported that miR-377-3p can exacerbate cerebral ischemia/reperfusion injury in MCAO/R rats [9]. Nevertheless, the specific mechanism of miR-377-3p in regulating ischemic stroke remains unclear.

Berberine (BBR) is a polycyclic compound found in the Coptis chinensis, and has been conventionally used in the treatment of gastrointestinal infections for its antibacterial properties. Currently, more researches have indicated that berberine also has anti-atherosclerotic, hypoglycaemic, lipid metabolic and anti-tumour effects [10], although the mechanisms have not been investigated in depth. Currently, many studies have nominated the neuroprotective effects of berberine. For example, berberine was detected to improve oxidative stress-induced damage in the brain of diabetic rats [11], and Huang et al. found that berberine has anti-inflammatory effects to protect brain damage in mice with traumatic brain injury [12]. It has also been demonstrated that berberine can exert pharmacological activity through several miRNAs. For example, berberine inhibited the migration and proliferation of endometrial cancer cells by miR-101 [13]. Berberine upregulates miR-340-5p to protect against HMGB1-mediated myocardial ischemia/reperfusion injury [14]. Above studies suggest that the natural drug berberine is a promising treatment for acute ischemic stroke. However, no research has clarified whether berberine improves ischemic stroke by regulating miR-377-3p.

LncRNAs are ncRNAs larger than 200 nt and play vital roles in a variety of processes including epigenetic regulation. Some LncRNAs bind miRNAs in the sponges-dependent manner, thus preventing miRNAs from binding to their target mRNAs [15]. Currently, various lncRNAs have been supposed to play important regulatory roles in brain I/R injury by mediating different signaling pathways. It has been determined that NEAT1 regulates the interaction between autophagy-associated proteins after neuronal I/R injury and alleviates neuronal reperfusion injury [16, 17]. This suggests that lncRNA NEAT1 is engaged in ischemic stroke, however the exact mechanism by which lncRNA NEAT1 regulates ischemic stroke remains to be explored.

N6-methyladenosine (m6A) modification, a dynamic reversible process of the adenosine N6 site, is the most typical epigenetic modification of mRNA post-transcriptional modifications, including the processes of methylation, demethylation and recognition, involving various protein molecules [18]. METTL3, METTL14 and WTAP are the main methylation transferase (writer), among which METTL3 is the most common methylation transferase whose action can be eliminated by the demethylases (erasers) FTO and ALKBH5. Then m6A recognition proteins (reader), including YTH structural domain family (YTHDF) proteins, further influence the nucleation and stability of mRNA. Recently, studies have indicated that m6A acts as a crucial factor in a range of diseases such as obesity, cancer, and viral infections [19]. A study demonstrated that overall m6A levels were altered throughout the brain transcriptome after stroke [20]. Studies revealed that m6A modification regulates the expression of genes involved in the stroke process [21]. Si et al. described that METTL3 promotes the maturation of miR-335 and reduces neuronal apoptosis [22], suggesting that METTL3-mediated m6A methylation alleviates IS. Notably, Wang et al. proved that induction of m6A in adipocyte exosomal LncRNAs mediated drug resistance in myeloma [23, 24]. This suggests that m6A modifications can regulate LncRNA function and stability to influence disease progression. However, whether m6A modification affects LncNEAT1 in ischemic stroke and the specific mechanisms have not been investigated.

In summary, we investigated whether berberine exerts neuroprotective effects on ischemic brain injury through the m6A methyltransferase METTL3 in mouse astrocytes, for which we simulated *in vitro* and *in vivo* models of IS. The study also provided diagnostic indicators and drug targets for treatment of IS.

## MATERIALS AND METHODS

### Animals and drug administration

Adult male C57BL/6J mice (weight 20–25 g; 8 weeks old) were gotten from Animal Model Center of Nanjing University. The above mice were placed under strictly controlled ambient temperature (22 ± 2° C) and relative humidity (50~60 %) and 12 h light/dark cycle (five per cage) allowed access to food and water. This study was approved by the Ethics Committee of Nanjing First Hospital (Approval Number of Ethics Committee: DWSY-23063367). All animal experiments were conducted in accordance with the ethical standards of Nanjing First Hospital.

Berberine (purity 98%) was gotten from Sigma (BP1108, USA). Given the present research, we randomly divided all mice into 3 groups, including sham, model (MCAO/R+Vehicle), and model (MCAO/R) + BBR (50 mg/kg). The dose and mode of BBR administration were determined according to our previous studies [25]. The BBR treatment group was pretreated with BBR (dissolved in 0.5% CMC-Na) by intragastric administered once a day for 14 consecutive days before the surgery. An equal dose of normal saline was administered to the other groups.

### Middle cerebral artery occlusion/reperfusion (MCAO/R) model mice

Firstly, mice were anesthetized with 2% isoflurane in O_2_ (RWD Life Science, Shenzhen, China). A neck incision was performed, exposing the right common carotid artery (CCA), external carotid artery (ECA), and internal carotid artery (ICA). After that, a silicon-coated monofilament (Cinontech, Beijing, China) was inserted into the CCA and advanced into the ICA, then, occluded the artery for 1h followed by reperfusion. The same surgical procedure was performed on sham group of mice, while without a monofilament put into the artery.

### Neurologic function scores

At 24 h after MCAO/R, neurological deficits of mice were assessed following the method described by Longa et al. [26]. The scoring criteria are as follows: 0, no deficit, normal walking; 1, mild deficit, fail to stretch forepaw; 2, moderate deficit, circling to the contralateral side; 3, severe deficit, falling to the contralateral side; 4, no spontaneous moving.

### 2,3,5-triphenyltetrazolium chloride (TTC) staining and infarct volume measurement

Triphenyl tetrazolium chloride (TTC) staining was performed at 24 h after surgery. Firstly, the brains were cut into 6 sections. Then, sections were stained using 2% TTC solution (Sigma-Aldrich, St. Louis, MO, USA) at 37° C for 0.5h, and fixed with 4% paraformaldehyde (Biosharp, Beijing, China). ImageJ software (National Institutes of Health, Bethesda, MD, USA) was used to calculate the percentages of normal tissue (red) and infarcted volume (white). The infarcted areas of each section were summed and presented as a percentage of the volume of un-infarcted areas: corrected percentage of infarct volume = [(contralateral hemisphere volume − ipsilateral non-infarct volume)/contralateral hemisphere volume] × 100%.

### Cell culture

Choosing the cerebral cortex of mice (1-3 days old) to extract primary astrocytes, the cortex was digested by 0.125% trypsin-EDTA (Gibco, Grand Island, NY, USA), then centrifuged and incubated in DMEM/F12 complete medium (Gibco, Thermo Fisher Scientific, Waltham, MA, USA). After 9-11 days, the cells were shaken at 260 rpm at 37° C for 16 h to purify. Finally, purified astrocytes were obtained.

Choosing the cortex of fetal mice (16–18 days old) to isolate primary neurons, the culture flasks were pretreated with poly-d-lysine (PDL) (Sigma-Aldrich, St. Louis, MO, USA). The fragment was digested with 0.125% trypsin and grown with DMEM (Gibco, Grand Island, NY, USA) for 5 h, then replaced with neurobasal medium containing 2% B27 (Gibco, Grand Island, NY, USA) and 0.5 mmol/L glutamine (Sigma-Aldrich, St. Louis, MO, USA).

Choosing Adult Brain Dissociation Kit and ACSA-2 MicroBeads (Miltenyi Biotec, Bergisch Gladbach, Germany) for magnetic isolation of astrocytes from adult mice. The former was used to digest the cortex. Then, purified astrocytes were obtained. Astrocytes were incubated for 15 min at 4° C with ACSA-2 MicroBeads and separated from single-cell suspension in a magnetic field using MS columns, MACS MultiStand and QuadroMACS (Miltenyi Biotec, Bergisch Gladbach, Germany).

### Primary astrocytes were co-cultured with primary neurons

The transwell co-culture system was established as described [27, 28]. After transfection for 48h, the medium was discarded and the primary astrocytes were washed by PBS. The primary neurons were then placed at the bottom of the transwell plate (Corning Company, Corning, NY, USA). After washing with PBS, primary astrocytes were put in the upper part of the transwell plate and separated from bottom by a semi-permeable membrane. The cells were incubated in two separate chambers.

### Oxygen-glucose deprivation/reoxygenation (OGD/R) treatment

Cells were incubated with or without BBR (10 μM) for 24 h, then exposed to OGD/R treatment. So as to mimic the environment of cerebral ischemia/reperfusion *in vitro*, cells exposed glucose and oxygen deprivation followed reoxygenation. First, cells were cultured with DMEM (Gibco, Grand Island, NY, USA) without glucose and FBS (Gibco, Grand Island, NY, USA) in a 95% N_2_ and 5% CO_2_ chamber for 4 h. Then the glucose-free DMEM medium was replaced with neurobasal medium complete medium or DMEM/F12 complete medium, and cultured in a 95% air and 5% CO_2_ incubator for 24 h. The final concentration of BBR in the OGD/R experiment was 10 μM.

### Cell transfection and lentivirus infection

Briefly, miR-377-3p mimic, miR-377-3p inhibitor, and respective negative control (NC mimic, NC inhibitor) were purchased from Shanghai GenePharma Company (Shanghai, China). According to the manufacturer’s protocol, transfected them to cells with Lipofectamine 3000 (Invitrogen, Carlsbad, CA, USA). Lentiviruses loaded with shRNA were obtained from Shanghai GeneChem Company (Shanghai, China), including overexpressed METTL3 (Lenti-METTL3), sh-METTL3, overexpressed NEAT1 (Lenti-NEAT1), sh-NEAT1 and their corresponding lentiviral scramble control shRNA or their corresponding overexpression negative control vector (Lenti-NC). Lentivirus infection was performed according to the manufacturer’s instructions. The sequences above are shown in Supplementary Table 1. After transfection or lentiviral infection, cells were treated with or without 10 μM of BBR and then subjected to subsequent OGD/R treatment to simulate the brain ischemia-reperfusion environment *in vitro*.

### Cell counting kit-8 (CCK-8)

Cell Counting Kit-8 (Dojindo, Kumamoto, Japan) was used to detect cell viability. Firstly, cells were treated with 10 μL CCK-8. Finally, after culture for 2 h at 37° C, the absorbance value at 450 nm was measured using a microplate reader (Thermo Fisher Scientific, Waltham, MA, USA). The survival ratio of cells per group was normalized to the control group, in which the survival ratio was regarded as 100%.

### Lactate dehydrogenase (LDH) assay

Cellular injury or death was detected by the release of lactate dehydrogenase (LDH) in the medium supernatant. First, collecting cells treated with OGD/R and transfected, put them suspended in 96-well plate, and then placed at 37° C for 0.5 h. Choosing a LDH assay kit (Sigma-Aldrich, St. Louis, MO, USA) to carry out the experiment. The absorbance of the supernatant at 490 nm was then recorded using a microplate reader. The LDH activity was calculated as (absorbance of sample hole-absorbance of the control hole) / (absorbance of the standard hole - absorbance of the standard blank hole).

### HE staining

24 h after MCAO/R, mice were anesthetized with 2% isoflurane in O_2_ (RWD Life Science, Shenzhen, China). After that normal saline (200 mL) and 4% paraformaldehyde (Biosharp, Beijing, China) (80 mL) were perfused into the heart. Brain tissue samples were fixed with 4% paraformaldehyde, dehydrated and embedded in paraffin. 4 μm slices were cut with a paraffin microtome and attached to the slide. Dewaxing by xylene and gradient dehydration by ethanol were adopted. At room temperature, hematoxylin staining solution was used for 5 min, followed by 1% ethanol hydrochloride for 30s differentiation. The ammonia water was added for 1 minute and then turn blue. They were rinsed with distilled water. Eosin staining solution was added, room temperature 2 min, distilled water washed, ethanol gradient decolorization. The xylene was allowed to permeate. Finally, the slide is sealed with a neutral adhesive. Changes were observed and photographed.

### Nissl staining

Similarly, the mice were anesthetized by 2% isoflurane in O_2_ (RWD Life Science, Shenzhen, China), brain tissue were fixed with 4% paraformaldehyde, dehydrated and embedded in paraffin. 4 μm slices were cut with a paraffin microtome and attached to the slide. Then carried out the Nissl staining assay according to the protocol of the Nissl staining kit (Solarbio, Nanjing, China). The results were viewed and photographed.

### Immunofluorescence

The isolated astrocytes or neurons were characterized by staining marker GFAP or Neun. Firstly, cells were put in 4% paraformaldehyde (Biosharp, Beijing, China) for 20 min. After that, put them in PBS containing 0.1% Triton X100 (Beyotime, Shanghai, China) for 20 min. Then incubated them with 5 % BSA (Biofroxx, Shanghai, China) for 1 h. After above, primary astrocytes were incubated with rabbit monoclonal antibody anti-GFAP (1:200; #80788S; CST, USA) and primary neurons were incubated with a rabbit monoclonal antibody anti-NeuN (1:200; #24307; CST) overnight at 4° C and then incubated the next day with goat anti-rabbit IgG H&L (Alexa Fluor® 488) (1:200; ab150077; Abcam, Cambridge, MA, USA) or goat anti-rabbit IgG H&L (Alexa Fluor® 594) (1:200; ab150080; Abcam) and incubated with DAPI (1:300, Cat. # C1002, Beyotime) to stain nuclei. Finally, the images were observed and photographed. The number of GFAP-positive cells or Neun-positive cells was calculated from randomly selected microscopic fields. Astrocyte or neuronal purity was presented as percentage of the number of the percentage of GFAP-positive or Neun-positive cells to the total cells of view, both around 90% or more.

### mRNA stability assay

The cells were planted in 6-well plates overnight. Cells were incubated with 5 μg/ml ActD (CST, Danvers, MA, USA) for 0, 1, 2, 4 h or longer. Then collected cells to extract total RNA, and target RNA was quantized by RT-qPCR for further analysis.

### Bioinformatic analysis

The possible miRNAs-related targets of Nampt were analyzed by online bioinformatics database miRDB, TargetScan and miRWalk. And through bioinformatics software RNAhybrid (https://bibiserv.cebitec.unibielefeld.de/rnahybrid/) to assess the combine about miR-377-3p with LncNEAT1. The bioinformatics software m6Avar (http://www.cuilab.cn/sramp/) was used to analyze the potential m6A sites on LncNEAT1.

### Dual-luciferase reporter gene assay

First, the luciferase reporter plasmids Nampt-WT and Nampt-Mut was constructed, then cells were co-transfected with above plasmids and NC mimic or miR-377-3p mimic respectively by using Lipofectamine 2000 (Invitrogen, Carlsbad, CA, USA). After co-incubation for 48h, cells were collected for lysis and luciferase activity was measured by the dual luciferase reporting and detection system (Promega, WI, USA). Similarly, the protocol also used to explore the combined effects of NEAT1 and miR-377-3p.

### Methylated RNA immunoprecipitation (MeRIP) assay

Firstly, Trizol method was used to extract total RNA. The RNA cracking reagent (AM8740, Invitrogen) was used to cut RNA into fragments. Anti-m6a antibodies (ab208577, Abcam) were incubated with RNA overnight at 4° C. Protein A/G Magnetic Beads (88803, Thermo Fisher Scientific, USA) was mixed with antibody treated RNA. A protease K buffer digested the m6A antibody. Finally, the methylated RNA was purified for RT-qPCR.

### Western blotting

Firstly, RIPA solution (Beyotime, Shanghai, China) was used to extract total proteins from mouse brain tissue or cells. Total protein concentration was measured and quantified using a BCA kit (Thermo Fisher Scientific, Grand Island, NY, USA). Protein samples mixed with 5×loading buffer were denatured in boiling water, separated by gel electrophoresis and transferred to PVDF membrane (Merck Group, Darmstadt, Germany). Closed with 5% skim milk for 1h and incubated with the following primary antibodies at 4° C overnight: rabbit monoclonal anti-Nampt (1:1000; 11776-1-AP; Proteintech, China), β-actin (1:1000; ab241153; Abcam), rabbit monoclonal anti-METTL3 (1:1000; 15073-1-AP; Proteintech). PVDF membrane is then paired with corresponding HRP labeled anti-Rabbit IgG (1:4000; #7074S; CST) was incubated at room temperature for 2h. Strip visualization was done using the ECL kit (Thermo Fisher Scientific, USA). The results were quantified using software ImageJ.

### Quantitative real-time polymerase chain reaction (RT-qPCR)

Trizol reagent (Thermo Fisher Scientific, USA) extracts total RNA from tissues or cells. RNA samples were reversely transcribed into cDNA using kits (ABM, Richmond, British Columbia, Canada). According to the standard SYBR-Green method, the ABI7500 type sequence detection system (7500, ABI, USA) was used for the detection. β-actin being an internal control of mRNA, and U6 was an internal reference of miRNA. The primers are shown in Supplementary Table 2.

### Statistical analysis

These experiments were performed at least three times. All values are expressed as mean ± standard deviation. Prism 8.0 (GraphPad Software, Inc., San Diego, CA, USA) should be used in all statistical analysis. All data were normally distributed. The difference between the two groups was checked by the T-test, and the comparison between more than two groups was checked by the analysis of variance. *P*<0.05 was regarded as statistically significant.

### Data availability statement

All data generated or analyzed during this study are available from the corresponding author upon reasonable request.

## RESULTS

### Berberine alleviates MCAO/R brain injury and up-regulates the expression of Nampt in adult mouse astrocytes

**Figure 1:**
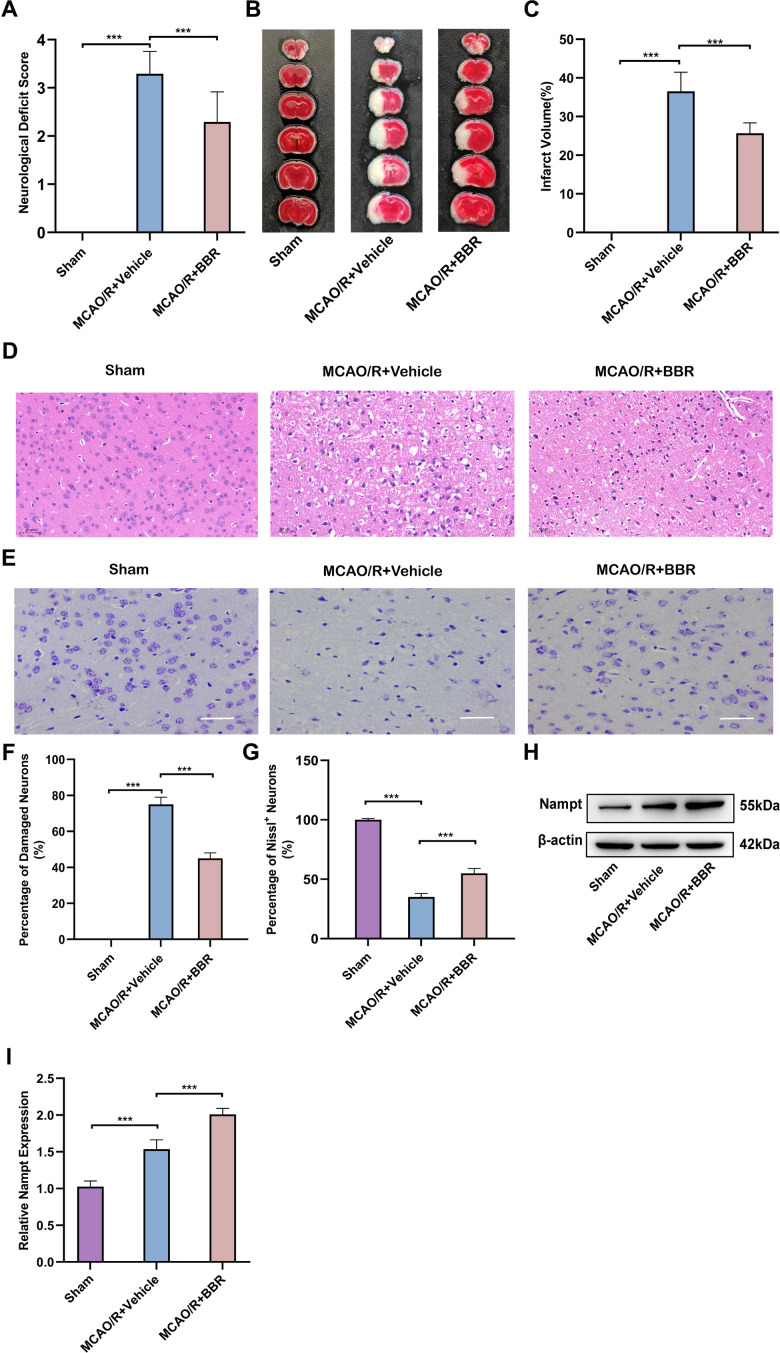
**Berberine alleviates MCAO/R brain injury and up-regulates the expression of Nampt in adult mouse astrocytes.** (**A**) Neurologic function scores for assessing the degree of neurological impairment after 24 hours in sham, MCAO/R+Vehicle or BBR+ MCAO/R group of mice (n=24). (**B**) Representative images of TTC-stained brain sections from mice (n=6). (**C**) Quantification of infarct volume at 24 h after MCAO/R (n=6). (**D**) Representative images of HE staining of ischemic brain tissue from mice (n=6, Scale bar: 50 μm). (**E**) Representative images of Nissl staining of ischemic brain tissue from mice (n=6, Scale bar: 50 μm). (**F**) Quantification of damaged neurons. (**G**) Quantification of Nissl+ neurons. (**H**) Representative Western blot images depicting Nampt in adult mouse astrocytes (n=6). (**I**) A bar presenting the quantification of Nampt (n=6). The relative expression levels were quantified by normalizing to β-actin. Data are represented as mean ± SD, (*P < 0.05; **P < 0.01; ***P < 0.001).

Firstly, the mice were put to MCAO/R to simulate cerebral I/R injury *in vivo*. Compared with the sham group, the nerve function defect and infarct volume of MCAO/R mice were strikingly increased, and the above results were reversed after administration of BBR (Figure 1A–1C). Meanwhile, HE staining and Nissl staining were used to observe the degree of brain histological damage in mice. The results indicated that ischemic stroke resulted in shrinkage of nucleus of neurons, widened pericellular spaces, as well as vacuolization in neuropil and decrease of Nissl bodies, while berberine administration relatively reduced brain damage in mice (Figure 1D–1G). Then we isolated astrocytes from 8-week-old adult mice. Western blot analysis demonstrated that compared with the sham group, the expression of Nampt in astrocytes of MCAO/R mice brain tissue was increase, while BBR administration was further increased the expression of Nampt (Figure 1H, 1I). To sum up, these results suggest that berberine may reduce the brain injury of mice after MCAO/R and act as a protective role against I/R injury by up-regulating the expression of Nampt in mouse astrocytes.

### miR-377-3p targets and inhibits the Nampt expression in OGD/R-treated primary astrocytes, thus promoting neuronal injury

**Figure 2:**
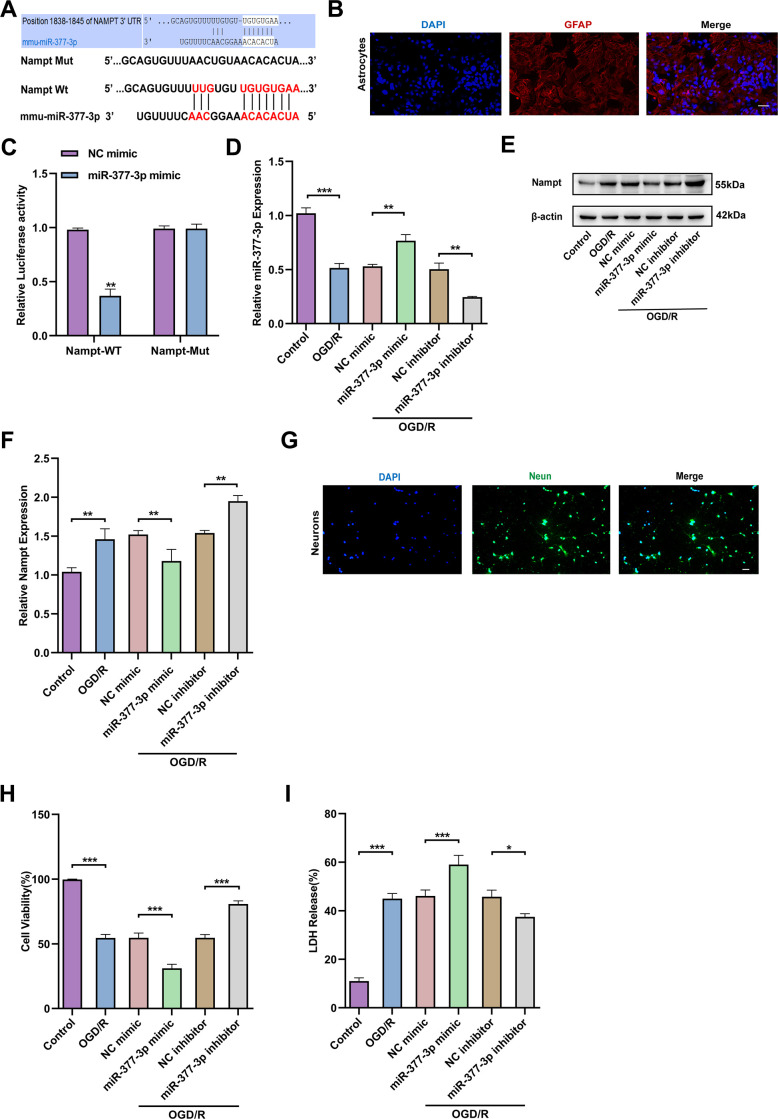
**miR-377-3p inhibits the expression of Nampt in astrocytes after OGD/R, thus promoting neuronal injury.** (**A**) Possible miRNA binding site on Nampt mRNA (predicted candidate miR-377-3p target gene by bioinformatics databases). (**B**) GFAP immunofluorescence validation of astrocytes (immunofluorescence images of primary astrocytes. Scale bar: 20 μm). (**C**) Relative luciferase activity of Nampt wild-type and 3ʹ-UTR mutant structures transfected with miR-377-3p mimics and NC mimic. (**D**) RT-qPCR detection of miR-377-3p levels in primary astrocytes after OGD/R following miR-377-3p mimic or miR-377-3p inhibitor treatment. (**E**) Western blot analysis of Nampt protein expression in primary astrocytes after OGD/R following miR-377-3p mimic or miR-377-3p inhibitor treatment. (**F**) A bar presenting the quantification of Nampt in primary astrocytes. (**G**) NeuN immunofluorescence validation of neurons (immunofluorescence images of primary neurons. (Scale bar: 20 μm.). (**H**) The co-cultured neuron viability was determined by CCK-8 assay after miR-377-3p mimic or miR-377-3p inhibitor transfection in astrocytes and OGD/R treatment. (**I**) LDH assay to detect the effect on co-cultured neurons after miR-377-3p mimic or miR-377-3p inhibitor treatment in primary astrocytes and OGD/R treatment. The relative expression levels were quantified by normalizing to β-actin. Data are represented as mean ± SD, (n = 3; *P < 0.05; **P < 0.01; ***P < 0.001).

Above three online bioinformatics databases were used to perform the potential miRNAs that can bind to 3’-UTR of Nampt were screened and miR-377-3p was initially determined (Figure 2A). Then, we extracted mouse primary astrocytes (Figure 2B). In order to further confirm the direct binding of Nampt and miR-377-3p, we conducted dual-luciferase reporter gene assay. As expected, miR-377-3p dramatically reduced the activity of Nampt-WT luciferase, but did not reduce the activity of Nampt-mut (Figure 2C). In order to further evaluate the interaction between Nampt and miR-377-3p, we silenced or over-expressed the level of miR-377-3p in primary astrocytes of mice and then treated by OGD/R (Figure 2D). The results showed that after OGD/R, compared with the corresponding control group, overexpression of miR-377-3p prominently reduced the level of Nampt, while knocking down miR-377-3p distinctly increased the level of Nampt (Figure 2E, 2F). To determine the influence of miR-377-3p in neuroprotective effects associated with Nampt, we extracted the primary neurons of mice (Figure 2G). After primary astrocytes were transfected with miR-377-3p mimic or miR-377-3p inhibitor, they were co-cultured with primary neurons, and then the system treated by OGD/R. CCK8 assay indicated that inhibition of miR-377-3p in primary astrocytes can promote the activity of its co-cultured neurons after OGD/R, while miR-377-3p mimic decreased the neuronal activity (Figure 2H). At the same time, the LDH experiment results also revealed that compared with the corresponding control group, the primary astrocyte transfected with miR-377-3p inhibitor attenuated the death of neurons co-cultured with it after OGD/R, while the transfection of miR-377-3p mimic increased the death of neurons (Figure 2I). Generally, our work confirm that the neuronal damage mediated by OGD/R-treated primary astrocytes is related to its Nampt/miR-377-3p signal transduction.

### Berberine regulates Nampt expression through downregulation of miR-377-3p in OGD/R-treated primary astrocytes, thereby affecting neuronal activity

**Figure 3:**
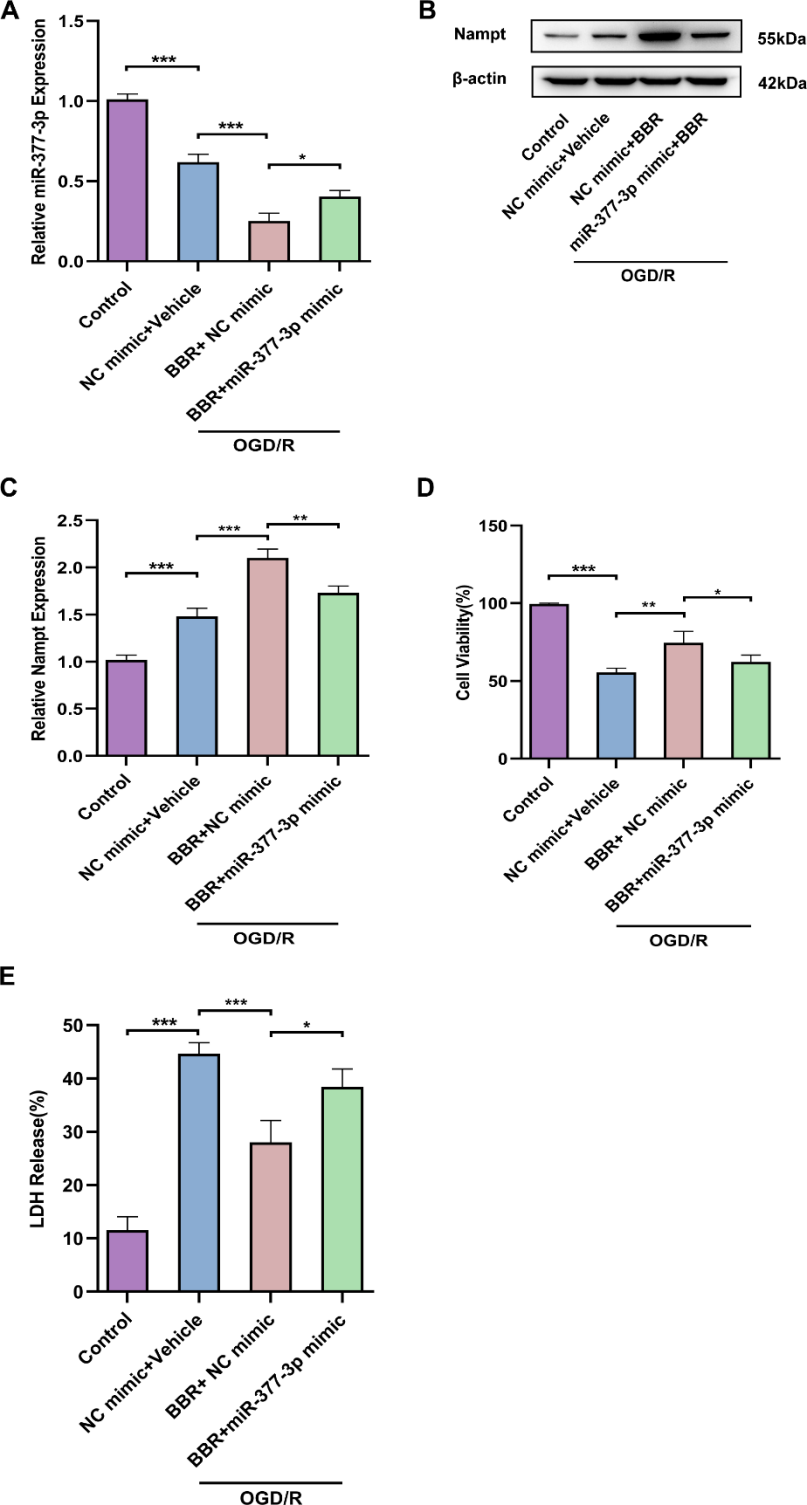
**Berberine regulates Nampt expression through downregulation of miR-377-3p in OGD/R-treated primary astrocytes, thereby affecting neuronal activity.** (**A**) Using RT-qPCR to analysis the miR-377-3p level in cells transfected with miR-377-3p mimic after berberine treatment. (**B**) Using Western blot to analyze Nampt expression in astrocytes after berberine treatment and transfection with miR-377-3p mimic. (**C**) A bar presenting the quantification of Nampt in primary astrocytes. (**D**) Using the CCK-8 assay to evaluate the effect of co-culture with berberine and primary astrocyte transfected with miR-377-3p mimic on neurons after OGD/R. (**E**) Using LDH assay to study the effect of co-culture with berberine and primary astrocyte transfected with miR-377-3p mimic on neurons after OGD/R. The relative expression levels were quantified by normalizing to β-actin. Data are represented as mean ± SD, (n = 3; *P < 0.05; **P < 0.01; ***P < 0.001).

Subsequently, we wanted to further investigate whether berberine plays a neuroprotective role by regulating Nampt-related pathways in primary astrocytes. RT-qPCR results nominated that miR-377-3p was down-regulated in primary astrocytes after OGD/R compared to control. Compared with the corresponding control group, miR-377-3p in OGD/R-induced primary astrocytes was further down-regulated after berberine treatment. Meanwhile, overexpression of miR-377-3p in OGD/R-treated primary astrocytes reversed the decrease of miR-377-3p induced by berberine (Figure 3A). Western blot analysis revealed that Nampt expression was up-regulated in post-OGD/R primary astrocytes. Compared with the corresponding control group, after berberine treatment, Nampt expression was further up-regulated in OGD/R-induced primary astrocytes, and overexpression of miR-377-3p in OGD/R-treated astrocytes reversed the up-regulation of Nampt expression induced by berberine (Figure 3B, 2C). Significantly, compared with the corresponding control group, berberine treatment can attenuate the neuronal damage induced by primary astrocytes after OGD/R. Nevertheless, transfection of miR-377-3p mimic reversed the neuroprotective effect induced by BBR treatment in primary astrocytes after OGD/R, which can be demonstrated by the decreased neuronal activity and increased LDH release (Figure 3D, 3E). In conclusion, berberine plays a neuronal protective role by down-regulating the expression of miR-377-3p through Nampt in primary astrocytes.

### NEAT1 binds to miR-377-3p as a ceRNA in primary astrocytes and plays a neuronal protective role

**Figure 4:**
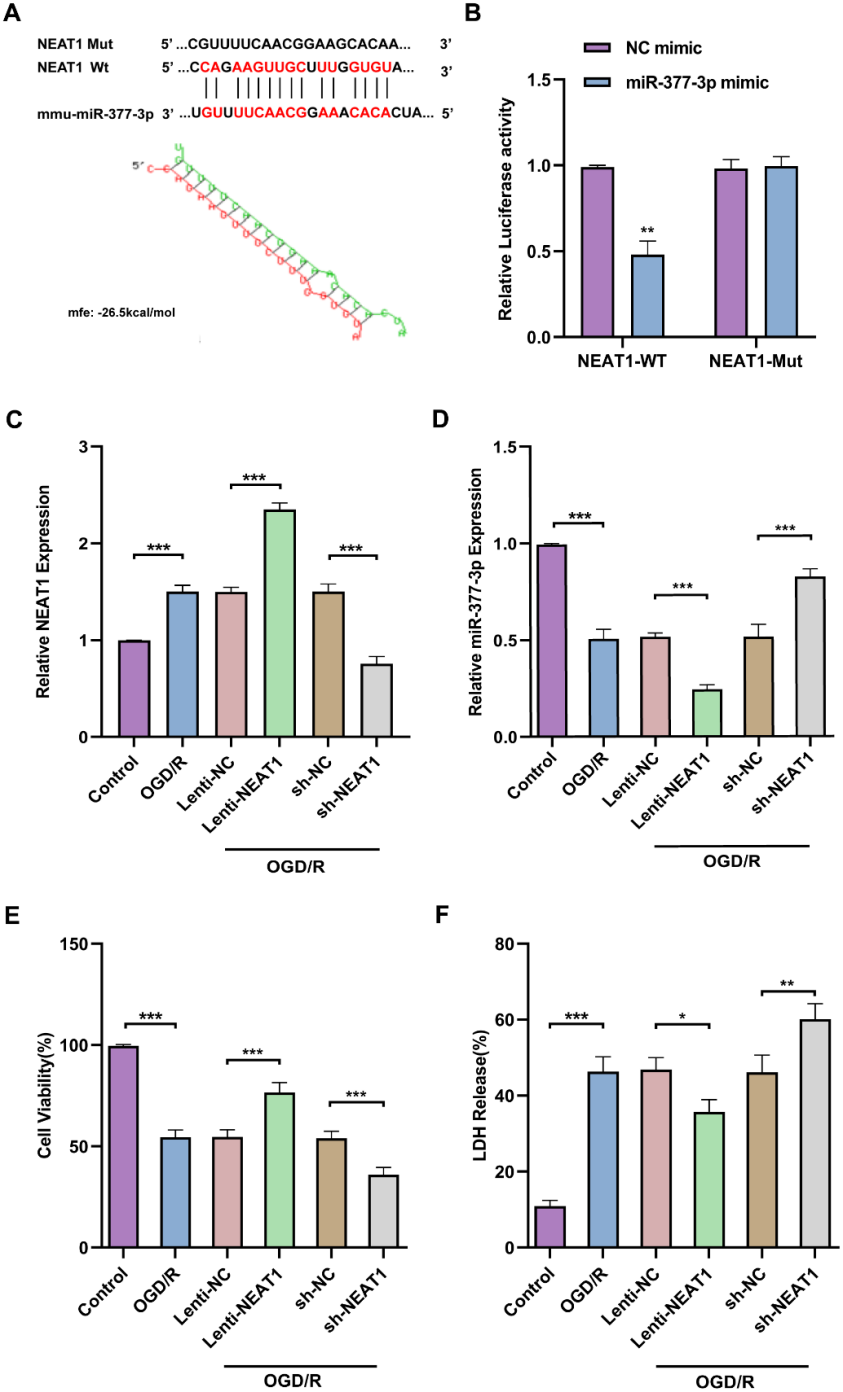
**NEAT1 binds to miR-377-3p in OGD/R astrocytes and plays a neuronal protective role as a ceRNA.** (**A**) Prediction of NEAT1 binding sites on miR-377-3p using bioinformatics software (RNAhybrid). (Schematic diagram of the sequence complementation relationship of miR-377-3p in NEAT1.) (**B**) Dual luciferase reporter gene analysis to confirm the binding relationship between NEAT1 and miR-377-3p. (**C**) RT-qPCR to analyze the NEAT1 level in primary astrocytes transfected with sh-NEAT1 or Lenti- NEAT1. (**D**) RT-qPCR to analyze the miR-377-3p level in primary astrocytes transfected with sh-NEAT1 or Lenti- NEAT1. (**E**) CCK-8 assay to assess the co-cultured neuron viability after NEAT1 knockdown or overexpression in primary astrocytes and OGD/R treatment. (**F**) LDH assay to study the effect of the co-cultured neuron viability after NEAT1 knockdown or overexpression in primary astrocytes and OGD/R treatment. Data are represented as mean ± SD, (n = 3; *P < 0.05; **P < 0.01; ***P < 0.001).

It is reported that LncNEAT1 can alleviate cerebral ischemia/reperfusion injury [29]. Firstly, the results assessed by the bioinformatics software RNAhybrid (https://bibiserv.cebitec.unibielefeld.de/rnahybrid/) indicated that miR-377-3p has a strong ability to bind to NEAT1 (Figure 4A). Meanwhile, the dual-luciferase reporter gene assay results confirmed that miR-377-3p-mimic markedly down-regulated the luciferase activity of NEAT1-WT, but did not down-regulate the luciferase activity of NEAT1-Mut (Figure 4B). In order to further evaluate the interaction between NEAT1 and miR-377-3p, we knocked-down or over-expressed the level of NEAT1 in primary astrocytes of mice and then treated by OGD/R (Figure 4C). Next, our results revealed that overexpression of NEAT1 significantly decreased the level of miR-377-3p in primary astrocytes, while knocking down NEAT1 prominently increased the level of miR-377-3p (Figure 4D). The primary astrocytes were knocked-down or overexpressed NEAT1 and co-cultured with the primary neurons, then the system treated by OGD/R. CCK8 assay demonstrated that compared with the corresponding control group, overexpression of NEAT1 in primary astrocytes promoted the activity of its co-cultured neurons exposed to OGD/R treatment, while knocking down NEAT1 weakened the neuronal activity (Figure 4E). At the same time, the results of LDH assay also indicated that compared with the corresponding control group, after OGD/R, the NEAT1 overexpression in primary astrocytes reduced the death of co-cultured neurons, while knocking down NEAT1 increased the death of neurons (Figure 4F). In general, the above results showed that NEAT1 binds to miR-377-3p as a ceRNA in primary astrocytes and plays a neuronal protective role.

### Berberine exerts neuroprotective effects through modulation of the NEAT1/ miR-377-3p /Nampt axis in primary astrocytes

**Figure 5:**
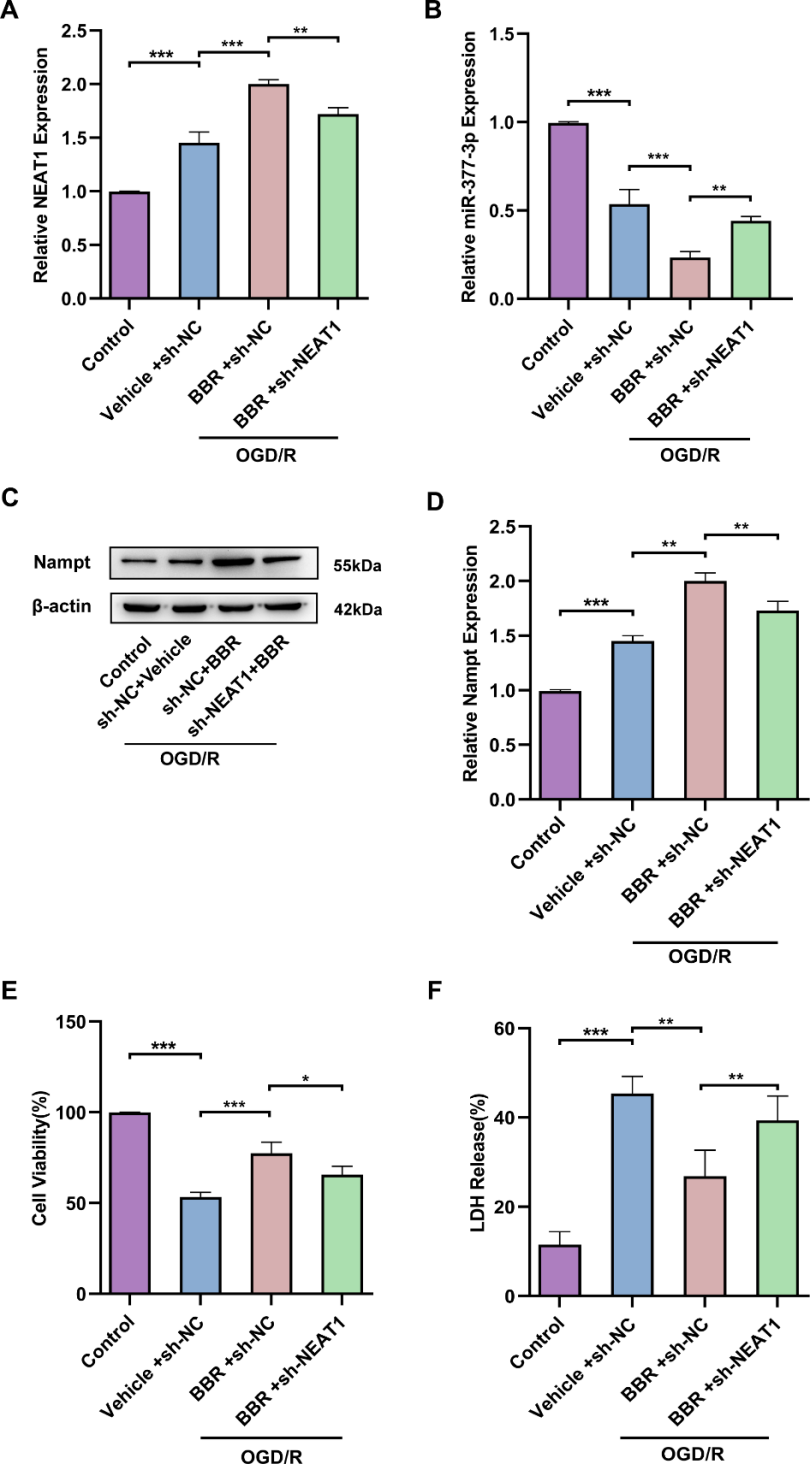
**Berberine exerts neuroprotective effects through modulation of the NEAT1/ miR-377-3p /Nampt axis in astrocytes.** (**A**) RT-qPCR to analyze the NEAT1 level in primary astrocytes transfected with sh-NEAT1 after berberine treatment. (**B**) RT-qPCR to analyze the miR-377-3p level in primary astrocytes transfected with sh-NEAT1 after berberine treatment. (**C**) Western blot analysis of Nampt expression in primary astrocytes transfected with sh-NEAT1 after berberine treatment. (**D**) A bar presenting the quantification of Nampt in primary astrocytes. (**E**) CCK-8 assay to assess the effect on co-cultured neurons by treating with NEAT1 knockdown and berberine-treated primary astrocytes and OGD/R treatment. (**F**) LDH assay to study the effect on co-cultured neurons by treating with NEAT1 knockdown and berberine-treated primary astrocytes and OGD/R treatment. The relative expression levels were quantified by normalizing to β-actin. Data are represented as mean ± SD, (n = 3; *P < 0.05; **P < 0.01; ***P < 0.001).

Next, we wanted to further explore whether berberine exerts neuroprotective effects by regulating NEAT1/miR-377-3p/Nampt-related pathway in primary astrocytes. RT-qPCR results confirmed that NEAT1 was upregulated in primary astrocytes after OGD/R compared to control, and NEAT1 was further upregulated in primary astrocytes after OGD/R following berberine treatment compared to the corresponding control group, while sh-NEAT1 transfection of primary astrocytes reversed the increase of NEAT1 expression induced by berberine treatment (Figure 5A). Meanwhile, the results nominated that miR-377-3p was downregulated in primary astrocytes after OGD/R following berberine treatment compared to the corresponding control group, while sh-NEAT1 transfection in primary astrocytes reversed the decrease in miR-377-3p induced by berberine treatment (Figure 5B). Western blot analysis revealed that Nampt expression was upregulated in primary astrocytes after OGD/R following berberine treatment compared to the corresponding control group, and sh-NEAT1 transfection of primary astrocytes reversed the increase in Nampt induced by berberine treatment (Figure 5C, 5D). Notably, transfection of sh-NEAT1 in primary astrocytes reversed the neuroprotective effect induced by berberine treatment compared to the corresponding controls, as evidenced by the decreased neuronal activity and increased LDH release (Figure 5E, 5F). Taken together, it can be known that LncRNA NEAT1 can be regarded as a competitive endogenous RNA (ceRNA) for miR-377-3p and block miR-377-3p-dependent target gene repression (i.e. Nampt). Concurrently berberine can upregulate NEAT1 and further regulate Nampt-related signaling in post-OGD/R primary astrocytes, thereby affecting neuronal activity.

### METTL3 enhances NEAT1 stability in primary astrocytes by modulating m6A modification of NEAT1

**Figure 6:**
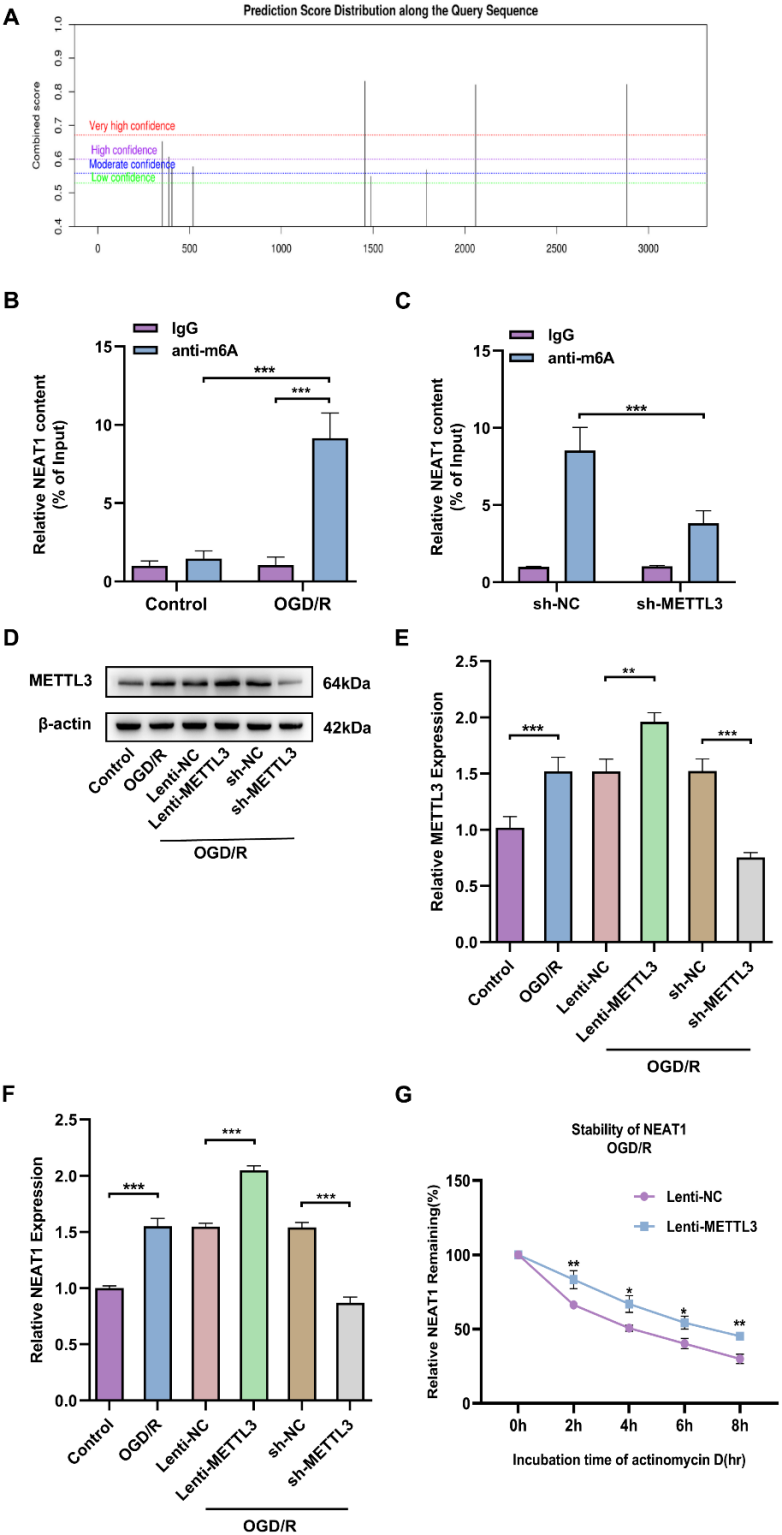
**METTL3 enhances NEAT1 stability in OGD/R astrocytes by modulating m6A modification of NEAT1.** (**A**) Prediction of potential m6A targets in Lnc NEAT1 based on SRAMP online website. (**B**) MeRIP-PCR to detect the m6A modifications in Lnc NEAT1. (**C**) Application of MeRIP-PCR to detect the correlation between NEAT1 and METTL3. (**D**) Western blot to detect expression of METTL3 after overexpression or silencing in primary astrocytes. (**E**) A bar presenting the quantification of METTL3 in primary astrocytes. (**F**) Expression of NEAT1 after METTL3 overexpression and silencing in primary astrocytes of mice after OGD/R. (**G**) Stability of NEAT1 in primary astrocytes with METTL3 overexpression after OGD/R treatment. Data are represented as mean ± SD, (n = 3; *P < 0.05; **P < 0.01; ***P < 0.001).

To probe the possible mechanisms about the regulation of neuronal protective effects by NEAT1, we examined the N6-methyladenosine (m6A) methylation of NEAT1. We used the bioinformatics software m6Avar to analyze potential m6A sites on LncNEAT1 and found multiple positions on LncNEAT1 with high confidence (Figure 6A). In addition, MeRIP-qPCR results confirmed that following OGD/R stimulation, LncNEAT1 m6A methylation levels were significantly increased in primary astrocytes (Figure 6B).

Published studies have revealed that methylation transferase METTL3 can increase NEAT1 expression by promoting m6A modification of NEAT1 [30]. We assessed the possibility that methyltransferase METTL3 is involved in regulating NEAT1 m6A modification in ischemic stroke by MeRIP-qPCR. The result indicated that knockdown of methyltransferase METTL3 apparently decreased m6A modification on NEAT1 (Figure 6C). Next, to further investigate the effect of METTL3 on NEAT1 expression levels, we knocked down or overexpressed METTL3 in primary astrocytes of mice and then treated with OGD/R (Figure 6D, 6E). Furthermore, RT-qPCR results revealed that the level of NEAT1 was significantly up-regulated in primary astrocytes after OGD/R treatment with overexpressing METTL3 and down-regulated in METTL3 knockdown cells compared to the corresponding controls (Figure 6F). To further certify the rationality of METTL3-mediated NEAT1 regulation, we investigated the effect of METTL3 overexpression on NEAT1 stability. Overexpressed of METTL3 in primary astrocytes significantly increased the stability of NEAT1 early after OGD/R (4h) upon ActD treatment (Figure 6G). The above findings reveal that METTL3 enhances NEAT1 stability through m6A modification in OGD/R-treated primary astrocytes.

### Berberine exerts neuroprotective effects via METTL3 regulating the NEAT1/miR-377-3p/Nampt axis in post-OGD/R primary astrocytes

**Figure 7:**
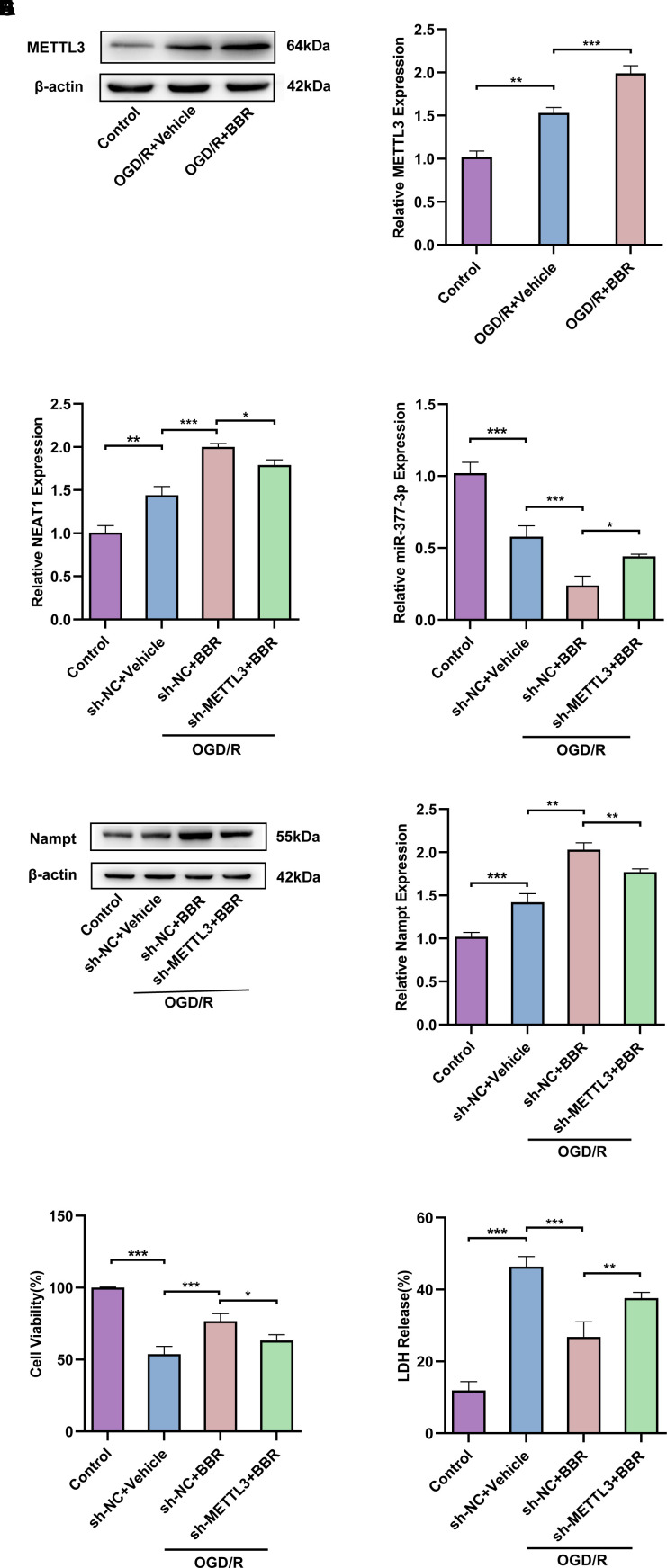
**Berberine exerts neuroprotective effects via METTL3 regulating the NEAT1/miR-377-3p/Nampt axis in post-OGD/R primary astrocytes.** (**A**) Western blot to detect expression of METTL3 in primary astrocytes after OGD/R and berberine treatment. (**B**) A bar presenting the quantification of METTL3 in primary astrocytes. (**C**) RT-qPCR to analysis the ex-pression of NEAT1 in primary astrocytes after OGD/R following berberine treatment and transfection with sh-METTL3. (**D**) RT-qPCR to analyze the expression of miR-377-3p in astrocytes after OGD/R following berberine treatment and transfection with sh-METTL3. (**E**) Western blotting to analyze the expression of Nampt in astrocytes after OGD/R following berberine treatment and transfection with sh-METTL3. (**F**) A bar presenting the quantification of Nampt in primary astrocytes. (**G**) Using CCK-8 assay to assess the effect on co-cultured neurons by treating with sh-METTL3 and berberine-treated primary astrocytes and OGD/R treatment. (**H**) Using LDH assay to evaluate the effect on co-cultured neurons by treating with sh-METTL3 and berberine-treated primary astrocytes and OGD/R treatment. The relative expression levels were quantified by normalizing to β-actin. Data are represented as mean ± SD, (n = 3; *P < 0.05; **P < 0.01; ***P < 0.001).

In view of the above studies on the neuroprotective role of berberine in regulating NEAT1-related pathways in post-OGD/R primary astrocytes, we further investigated the effect of berberine on m6A modification in an *in vitro* model of ischemic stroke. Western blot results showed that METTL3 expression was upregulated after OGD/R compared with control group, besides, METTL3 expression was further upregulated in post-OGD/R primary astrocytes after berberine treatment compared with OGD/R group (Figure 7A, 7B). Meanwhile, knockdown of METTL3 in primary astrocytes after OGD/R reversed the increase of NEAT1 induced by berberine treatment (Figure 7C), but conversely made miR-377-3p expression up-regulated (Figure 7D). In contrast, compared with the corresponding control group, knockdown of METTL3 in primary astrocytes after OGD/R reversed the increase of Nampt induced by berberine treatment (Figure 7E, 7F). Notably, knockdown of METTL3 also reversed the neuronal protective effects induced by berberine treatment on OGD/R primary astrocytes compared to the corresponding controls, as evidenced by the relative reduction in neuronal viability and increased LDH release (Figure 7G, 7H). Taken together, we can know that berberine exerts neuroprotective effects via METTL3 regulation of the NEAT1/miR-377-3p/Nampt axis in post-OGD/R primary astrocytes.

### Berberine regulates METTL3-mediated m6A modification of NEAT1 to alleviate ischemic stroke in adult mouse astrocytes

**Figure 8:**
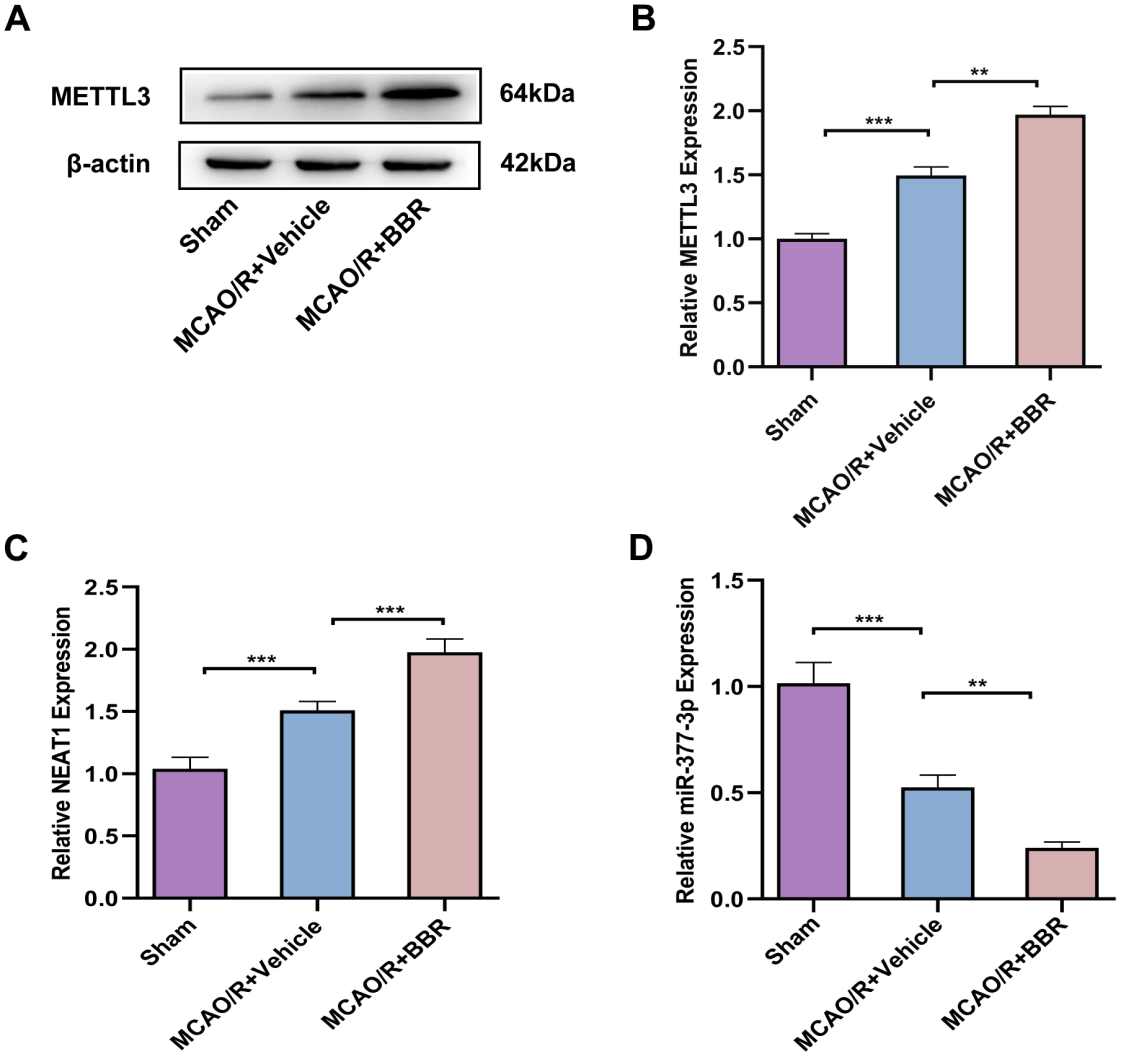
**Berberine regulates METTL3-mediated m6A modification of NEAT1 to alleviate ischemic stroke in adult mouse astrocytes.** (**A**) Western blot to verify METTL3 expression in adult mouse astrocytes after berberine administration following MCAO/R. (**B**) A bar presenting the quantification of METTL3. (**C**) RT-qPCR for NEAT1 expression in adult mouse astrocytes after berberine administration following MCAO/R. (**D**) RT-qPCR to verify the expression of miR-377-3p in adult mouse astrocytes after berberine administration following MCAO/R. The relative expression levels were quantified by normalizing to β-actin. Data are represented as mean ± SD, (n = 6; **P < 0.01; ***P < 0.001).

Finally, in view of the above experiments, we demonstrated the neuronal protective effect of berberine in an *in vitro* model of cerebral I/R and the specific correlated mechanism, so we wanted to further validate the neuronal protective effect mechanism of berberine in mice *in vivo*. Then we isolated astrocytes from 8-week-old adult mice. Western blot analysis confirmed that the expression of METTL3 was upregulated in astrocytes of brain tissue from MCAO/R mice compared with the sham group, and compared with the model group, BBR administration further increased METTL3 expression (Figure 8A, 8B). Meanwhile, RT-qPCR results further showed that the level of NEAT1 was upregulated in astrocytes of brain tissue from MCAO/R mice compared with the sham group, and further increased by BBR treatment (Figure 8C). In contrast, miR-377-3p levels were downregulated in astrocytes of brain tissue from MCAO/R mice compared to the sham group, and miR-377-3p expression further decrease by BBR administration compared to the model group (Figure 8D). These findings are consistent with the above *in vitro* results, suggesting that berberine exerts neuroprotective effects by regulating NEAT1/ miR-377-3p/Nampt axis in mouse astrocytes through METTL3, thereby alleviates ischemic stroke.

## DISCUSSION

Ischemic stroke is one of the principal reasons of disability and death globally. Both of Nampt levels in the body circulation and the brain are upregulated during acute cerebral I/R stress [31]. Nampt is a regulator of the intracellular NAD pool in mammalian cells. In recent years, several studies have identified that Nampt affects the ischemic stroke process through biological signaling or energy metabolism pathways, exerting roles including neuroprotection, vascular repair and neurogenesis. For example, Nampt prevents IS by rescuing neurons apoptosis through the sirt1-dependent AMPK pathway [32]. Secreted Nampt has a novel neuroprotective role in protecting white matter after ischemic injury [33]. Nampt-nicotinamide adenine dinucleotide cascade promotes regenerative neurogenesis after ischemic stroke [34]. Meanwhile, our previous study nominated that the astrocyte-derived exosome Nampt improves cerebral I/R injury by targeting autophagy associated signaling pathways AMPK/mTOR [35]. During the present research, Nampt expression was proved to be upregulated in astrocytes of a mouse brain ischemia/reperfusion model, and further upregulation of Nampt expression attenuated ischemia/reperfusion injury, while in an *in vitro* cellular model, Nampt also had a neuroprotective effect. These *in vitro* and *in vivo* results suggest that Nampt is a crucial target for the prevention and therapy of ischemic stroke.

Various miRNAs have been proved to indirectly regulate the nervous system immune response, oxidative stress and so on by affecting the expression of target mRNA, which influence the pathophysiology during IS. For instance, it is suggested that miR-155 promotes neuroinflammatory responses, endothelial activation in IS by targeting SOCS-1 [36]. miR-124 protects neurons and suppresses neuroinflammation in ischemic stroke by targeting Akt signaling pathways [37]. It is believed that miR-377-3p has been involved in inhibiting the survival and angiogenesis of cerebral microvascular endothelial cells (BMECs) induced by OGD treatment [38]. Meanwhile, Nampt promotes post-ischemia angiogenesis by modulating Notch signaling through the NAD+-SIRT1 cascade [39]. In this study, miR-377-3p was identified to be associated with Nampt, a therapeutic target in ischemic stroke, by bioinformatics software screening and luciferase reporter gene assay. Further experiments demonstrated that miR-377-3p inhibited Nampt expression in post-OGD/R astrocytes thereby promoting neuronal injury.

Berberine is widely used in clinical practice as an important active ingredient of the Chinese medicine Huang Lian. Several researches have verified that berberine can play a role in several diseases by regulating miRNAs. There is report that berberine had protective effects against vascular dementia in diabetic rats, and the effects depended on the downregulation of miR-133a [40]. Berberine attenuated neonatal sepsis in mice by inducing miR-132-3p to inhibit FOXA1 and NF-κB signaling [41]. Meanwhile, our previous findings found that berberine attenuated the inflammatory response to IS via the lncRNA Malat1/miR-181c-5p/HMGB1 axis [25]. According to present findings, we nominated that berberine exerts neuroprotective effects by downregulating miR-377-3p to regulate Nampt expression in post-OGD/R astrocytes.

miRNAs often interact with and negatively regulate molecular sponges of long-stranded non-coding RNAs, and many LncRNAs have been proved that differentially expressed in IS. LncRNA Malat1 has been reported to be upregulated and to promote ischemic stroke injury [42]. Meanwhile, LncRNA RMST has been reported to bind to miR-377-3p and act as ceRNA, activating SEMA3A-related signaling pathway, thereby exacerbating neuronal apoptosis in OGD/R-induced IS [43]. Our research suggest that LncNEAT1 may also be an underlying target of miR-377-3p in primary astrocytes of mice, for which NEAT1 combines with miR-377-3p and acts as a ceRNA to exert neuronal protection in IS.

m6A is the most common epigenetic modification of RNA, frequently found in the mammalian central nervous system, and ischemic stroke could alter the brain m6A transcriptome. METTL3 and YTHDC1 promote Akt phosphorylation to alleviate ischemic stroke by destabilizing PTEN mRNA [44]. m6A methylation has also been shown to inhibit the translation of the target gene p65 to avoid IS-induced inflammatory responses, all of which primarily via miR-421-3p targeting of m6A reader YTHDF1 [45]. LncRNAs function mainly by interacting with proteins or RNA through interaction sites contained in their sequences. The functional implementation of LncRNAs is related to m6A modification. On the one hand, the epigenetic mechanism of LncRNA associated with m6A modification further regulates the function and interaction of LncRNA by altering the structure of LncRNA, which plays an important role in the development diagnosis and treatment of many diseases. For example, Methyltransferase METTL3 directly mediates the m6A modification of LNCC00958 to enhance its stability, and then promotes the progression of hepatocellular carcinoma by up-regulating the expression of HDGF [46]. In an *in vitro* model of ischemia/reperfusion injury, METTL3 promotes miR-422a accumulation by inducing Lnc-D63785 m6A methylation [47]. METTL3 has been reported to target and enhance NEAT1 expression in macrophages [48]. Similarly, the m6A modification of LncRNA SNHG17 also enhances its stability and promotes lung adenocarcinoma resistance [49]. On the other hand, it has been reported that LncRNA miR4458HG binds to m6A reader IGF2BP to promote the stability of IGF2BP2-mediated target mRNA (HK2), thereby altering the physiology of human hepatocellular carcinoma [50]. KLF4 could increase m6A eraser FTO levels to decrease the m6A methylation of Drp1 by targeting lncRNA-ZFAS1, thus resulting in an inhibitory effect in IS-induced neuronal injury [51]. Here, we demonstrated that METTL3 acts as a “writer” for m6A modification and induces m6A modification on LncNEAT1, promoting its stability, thereby resulting in alleviation of ischemia/reperfusion injury.

It should be noted that this study has the limitation. Theoretically, berberine has low solubility and high molecular weight, for which it could not be absorbed into the blood circulation in large quantities, and its blood-brain barrier transmission rate is not high [52]. Our previous series of studies demonstrated that berberine improves ischemic stroke by attenuating the neuroinflammation and promoting angiogenesis [25]. All these studies suggest that berberine, which partially crosses the blood-brain barrier, can exert direct neuroprotective effects in ischemic stroke. Berberine has been reported to remain in the gut after entering the body and interact with the gut microbiota. Ischemic stroke can change the composition of the gut microbiota [53]. Meanwhile, gut microbiota can modulate the outcome of stroke and play a role in its development [54]. Wang et al. found that berberine attenuated ischemia-reperfusion injury in mice by modulating gut microbiota [55]. Therefore, the neuroprotective effect of berberine gavage on ischemic stroke may be indirectly achieved by modulating the gut microbiota, and its specific mechanism needs to be further explored.

## CONCLUSIONS

**Figure 9:**
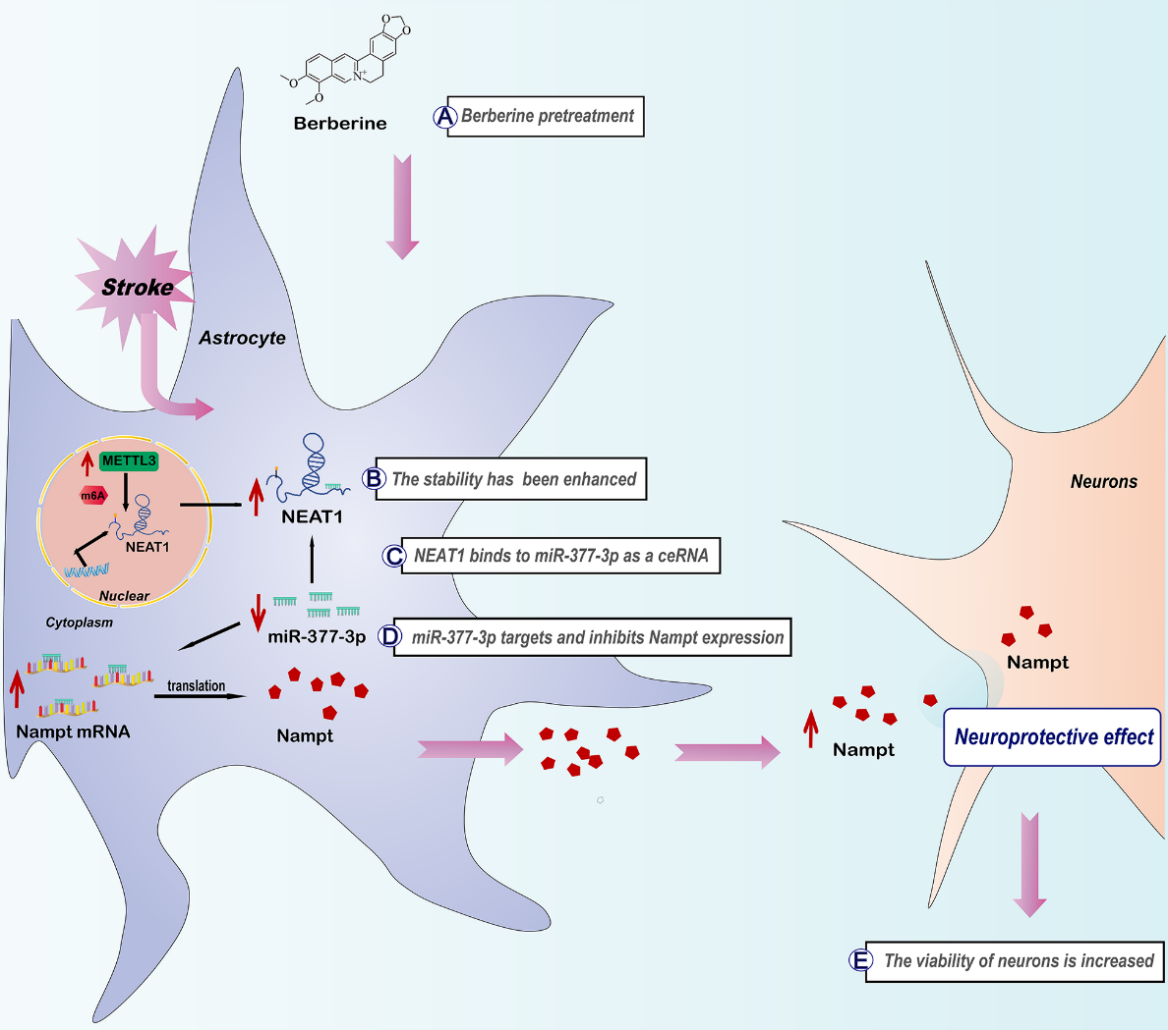
The schematic diagram of molecular mechanism in modulating METTL3-mediated m6A modification of NEAT1 in ischemic stroke by berberine via NEAT1/miR-377-3p/Nampt axis.

Our results confirm that the pharmacological activity of berberine in alleviating ischemic stroke is associated with Nampt, and reveal the associated mechanism of METTL3 and NEAT1 from the viewpoint of m6A modification. Mechanistically, after berberine administration, METTL3 can enhance the stability of NEAT1 via m6A modification, which in turn activates miR-377-3p/Nampt to exert neuroprotective effect (Figure 9). Collectively, the present findings show that berberine exerts neuroprotective effects via the m6A methyltransferase METTL3, which regulates the NEAT1/miR-377-3p/Nampt axis in mouse astrocytes to ameliorate cerebral ischemia/reperfusion injury.

## Supplementary Material

Supplementary Tables
