# Supplementary material for: METTL3-dependent N6-methyladenosine modification is involved in berberine-mediated neuroprotection in ischemic stroke by enhancing the stability of NEAT1 in astrocytes
Source: Aging (Albany NY). 2024 Jan 4;16(1):299–321. doi: 10.18632/aging.205369 (PMC10817396; doi:10.18632/aging.205369)
Supplement: Supplementary Tables [file aging-16-205369-s001.pdf]

SUPPLEMENTARY TABLES

Supplementary Table 1. The shRNA, miR-mimic and miR-inhibitor sequences.

Name	Sequence
sh-NC	5’-GATCCGCAGATGAAGGCACGGTCACGCTCGAGGCAGATGAAGGCACGGTCACGTTTTTG-3’<br>5’-AATTCAAAAAGCAGATGAAGGCACGGTCACGCTCGAGGCAGATGAAGGCACGGTCACGG-3’
sh-METTL3	5’-CACCGGACCAAGGAAGAGTGCATGACGAATCATGCACTCTTCCTTGGTCC-3’<br>5’-AAAAGGACCAAGGAAGAGTGCATGATTCGTCATGCACTCTTCCTTGGTCC-3’
sh-NEAT1	5’-CACCGGAGGAATCTTCCTTAGATGGCGAACCATCTAAGGAAGATTCCTCC-3’<br>5’-AAAAGGAGGAATCTTCCTTAGATGGTTCGCCATCTAAGGAAGATTCCTCC-3’
mmu-miR-377-3p inhibitor	ACAAAAGTTGCCTTTGTGTGAT
NC inhibitor	CAGUACUUUUGUGUAGUACAA
NC mimic	UUCUCCGAACGUGUCACGUTT
mmu-miR-377-3p mimic	ATCACACAAAGGCAACTTTTGT

Supplementary Table 2. Primer sequences for RT-qPCR.

Name	Sequence
mmu-NEAT1	F: 5’- AGGAGAAGCGGGGCTAAGTA-3′<br>R: 5’- TAGGACACTGCCCCCATGTA-3′
mmu-miR-377-3p	F: 5’- AGAGGTTGCCCTTGGTGAA-3′<br>R:5’-ACAAAAGTTGCCTTTGTGTG-3′
mmu-β-actin	F: 5’- CCACCATGTACCCAGGCATT-3′<br>R: 5’- CGGACTCATCGTACTCCTGC-3′
mmu-U6	F: 5’-CAGCACATATACTAAAATTGGAACG-3′<br>R: 5’-ACGAATTTGCGTGTCATCC-3′
